# Basal and dynamic relationships between serum anti-Müllerian hormone and gonadotropins in patients with functional hypothalamic amenorrhea, with or without polycystic ovarian morphology

**DOI:** 10.1186/s12958-022-00961-y

**Published:** 2022-07-04

**Authors:** Marlene Hager, Johannes Ott, Julian Marschalek, Marie-Louise Marschalek, Clemens Kinsky, Rodrig Marculescu, Didier Dewailly

**Affiliations:** 1 Clinical Division of Gynecological Endocrinology and Reproductive Medicine, Department of Obstetrics and Gynecology, Medical University of Vienna, Spitalgasse 23, 1090 Vienna, Austria; 2 Department of Obstetrics and Gynecology, Klinik Floridsdorf, Vienna, Austria; 3 Department of Laboratory Medicine, Medical University of Vienna, Vienna, Austria; 4 Faculty of Medicine Henri Warembourg, University of Lille, 59045 Lille, Cedex France

**Keywords:** Hypogonadotropic hypogonadism, Gonadotropin releasing hormone, Pulsatile GnRH treatment, GnRH stimulation test, Polycystic ovary syndrome

## Abstract

**Background:**

To evaluate in women with functional hypothalamic amenorrhea (FHA), whether there is a difference between patients with and without polycystic ovarian morphology (PCOM) concerning the response to a gonadotropin releasing hormone (GnRH) stimulation test and to pulsatile GnRH treatment.

**Methods:**

In a retrospective observational study, 64 women with FHA who underwent a GnRH stimulation test and 32 age-matched controls without PCOM were included. Pulsatile GnRH treatment was provided to 31 FHA patients and three-month follow-up data were available for 19 of these.

**Results:**

Serum levels of gonadotropins and estradiol were lower in FHA women than in controls (*p* < 0.05). FHA patients revealed PCOM in 27/64 cases (42.2%). FHA patients without PCOM revealed lower anti-Müllerian hormone (AMH) levels than controls (median 2.03 ng/mL, IQR 1.40–2.50, versus 3.08 ng/mL, IQR 2.24–4.10, respectively, *p* < 0.001). Comparing FHA patients with and without PCOM, the latter revealed lower AMH levels, a lower median LH increase after the GnRH stimulation test (240.0%, IQR 186.4–370.0, versus 604.9%, IQR 360.0–1122.0; *p* < 0.001) as well as, contrary to patients with PCOM, a significant increase in AMH after three months of successful pulsatile GnRH treatment (median 1.69 ng/mL at baseline versus 2.02 ng/mL after three months of treatment; *p* = 0.002).

**Conclusions:**

In women with FHA without PCOM, the phenomenon of low AMH levels seems to be based on relative gonadotropin deficiency rather than diminished ovarian reserve. AMH tended to rise after three months of pulsatile GnRH treatment. The differences found between patients with and without PCOM suggest the former the existence of some PCOS-specific systemic and/or intra-ovarian abnormalities.

**Supplementary Information:**

The online version contains supplementary material available at 10.1186/s12958-022-00961-y.

## Background

Functional hypothalamic amenorrhea (FHA) is considered to be due to chronic anovulation without identifiable organic causes [1]. It has its direct origin in a functional decrease or stop of pulsatile Gonadotropin Releasing Hormone (GnRH) secretion, which results in insufficient ovarian function [1] and is thought to be restorable when causative behavioural factors are corrected [2, 3]. In a very recent study on FHA and controls, Makollé et al. demonstrated that the serum levels of anti-Mullerian hormone (AMH) were significantly lower in FHA patients without polycystic ovarian morphology (PCOM) than in controls [4]. AMH, a glycoprotein which is secreted by the granulosa cells of primary and secondary follicles, is an important player in the regulation of follicle recruitment and growth [5]. Its serum level is considered as a good reflection of the ovarian reserve, i.e., the number of remaining primordial follicles [6]. The above-mentioned finding led the authors to the essential clinical suggestion that a low ovarian reserve should not be diagnosed on the basis of a decreased AMH level alone in women with FHA [4]. Moreover, the study showed once more that a relevant rate of women with FHA reveal PCOM, characterized by a high number of many small antral follicles at ultrasound and increased AMH levels compared to controls and non-PCOM FHA patients [4], which was in line with previous findings [7, 8]. It has been suggested that these peculiar patients initially had had components of polycystic ovary syndrome (PCOS) before losing weight and acquiring FHA. A higher body mass index (BMI) along with lower levels of sexual hormone binding globulin (SHBG) have been considered suggestive for this hypothesis [4].

Accordingly, one could forecast that women with FHA and PCOM would be prone to “re-develop” PCOS after restoration of their hypothalamic function which could then lead to a higher risk for staying anovulatory and, thus, amenorrhoic. However, a retrospective analysis has already suggested that this would not be the case. In contrast, the use of the pulsatile GnRH therapy led to highly satisfying results in this group of patients [9]. Since it has been reported that the prevalence of PCOS characteristics in unselected populations is much higher than the prevalence of clinically evident PCOS [10], a high rate of undetected cases can be assumed. Many of these seem to remain “incognito”, since they have regular ovulatory cycles and do not suffer from clinical manifestations of hyperandrogenism. Notably, women with PCOS show marked increases in the serum level of luteinizing hormone (LH) but not follicle stimulating hormone (FSH) after a short term stimulation test with Gonadotropin releasing hormone (GnRH) [11]. Similarly, higher LH levels after a GnRH test were found for regularly cycling ovulatory women with PCOM compared to normal ovulatory women without PCOM, despite the fact that patients fulfilling PCOS criteria still revealed even higher increases in LH [12].

The GnRH stimulation test has been considered a useful tool in the diagnosis of FHA [13]. Of note, despite the miscellaneous definitions of FHA which have been used in clinical studies, the GnRH stimulation test has been used only rarely [3]. This is in line with recent recommendations [14]. Nonetheless, in the last two decades, the test has been used at our department for women who wanted a reliable confirmation that their pituitary function would not be impaired in terms of LH and FSH production. In this study, we provide data about the stimulation GnRH test in a retrospective cohort of female FHA patients with and without PCOM compared to young healthy controls with neither FHA nor PCOM. In addition, we present available data from pulsatile GnRH treatment. Our results might provide new insights into the hormonal profile of FHA.

## Methods

### Patient population

This retrospective case–control study was conducted at the Clinical Division of Gynaecologic Endocrinology and Reproductive Medicine of the Medical University of Vienna, Austria, which serves as a referral centre for gynaecologic endocrinology and reproductive medicine. From January 2017 – December 2020, 64 women with FHA aged 16–38 years underwent a GnRH stimulation test. To offer this test to women with the suspicion of FHA has been a routine procedure at the department with the aim of providing a reliable confirmation of a normal pituitary capacity to produce LH and FSH. FHA was defined based on the following criteria: secondary amenorrhea for at least six months in a context of weight loss and/or insufficient caloric intake and/or intense physical activity and/or notion of recent psychological stress (see below); a negative progestin test with oral dydrogesterone 10 mg twice a day for ten days; exclusion of pregnancy, hypothyroidism and hyperprolactinemia; and exclusion of an organ-related pituitary cause by the use of a pituitary MRI.

In detail, all women with FHA reported fairly regular menstrual cycles prior to their complaint of amenorrhea. A weight loss > 10 kg prior to the onset of amenorrhea was rated as significant. A body mass index (BMI) < 18.5 kg/m^2^ according to the criteria for underweight was considered suggestive for FHA due to underweight [15]. Eating disorders were diagnosed according to the ICD-10 criteria [16]. Every “exercising” subject exercised at least 10 h per week, which included any type of exercise (dancing, aerobics, biking, etc.) or ran at least 30 miles per week [17]. History of emotionally stressful events preceding the onset of amenorrhea included problems within the family, at school, at work or of psychosocial stress (psychiatric diseases were excluded using DSM IV criteria) [18]. None of the women had hirsutism or acne.

In the control group, 32 age-matched women (aged 16–38 years) with regular menstrual cycles between 26 and 30 days were included. These women had originally been included as healthy controls in two prospective studies that have been recruited at the department. One has already been published [19]. Exclusion criteria for this group were LH < 2 IU/l, presence of PCOS according to the revised Rotterdam criteria [20] as well as the finding of PCOM on ultrasound (an antral follicle number > 12 and/or an ovarian volume 10 cm^3^ and/or an ovarian area 5.5 cm^2^) according to the recent reference study by Makollé [4], suspected low ovarian reserve with a FSH > 12 IU/l or hyperprolactinemia.

The study was approved by ethics committee of the Medical University of Vienna (IRB number 2436/2020, approved on April 21^st^ 2021).

### Parameters analysed

The AKIM-software (SAP-based patient management system at the Medical University of Vienna) was used for data acquisition. The main outcome parameters were serum levels of AMH, LH, and FSH. In addition, serum levels of total testosterone, androstenedione, Sex Hormone Binding Globulin (SHBG), prolactin and estradiol were also analysed. Blood samples were obtained during the early follicular phase visit (cycle days 2–5) in the controls and at any time in patients with FHA. All serum parameters were determined at the Department of Laboratory Medicine, Medical University of Vienna, according to ISO 15189 quality standards. As reported previously [21–23], Cobas electrochemiluminescence immunoassays (ECLIA) were performed on Cobas e 602 analysers (Roche, Mannheim, Germany) for the determination of serum estradiol, follicle-stimulating hormone (FSH), luteinizing hormone (LH), anti-Mullerian hormone (AMH), testosterone, dehydroepiandrosterone-sulphate (DHEA-S), thyroid-stimulating hormone (TSH) and sex hormone binding globuline (SHBG). The corresponding maximal coefficients of variation of these assays were 10.6, 4.5, 2.2, 3.5, 14.5, 2.7, 11.9 and 4.0%, respectively. Serum androstenedione concentrations were measured with the Liaison Androstenedione chemiluminescence immunoassay (CLIA) on Liaison XL analysers (DiaSorin, Saluggia, Italy) with a maximal coefficient of variation of 12.0%.

For the GnRH stimulation test, a bolus of 0.1 mg gonadorelin (Relefact ®, Sanofi-Aventis Germany GmbH, Frankfurt am Main, Germany) was given intravenously. Blood samples were drawn for the determination of serum LH and FSH levels just before (“baseline”) and 20 min (“post-stimulatory”) after the GnRH injection.

Age and BMI were collected as additional parameters as well as the follicle number per ovary (FNPO) on ultrasound. The vaginal ultrasound was performed on the same day as the blood retrieval with an “Aloka Prosound 6” ultrasound machine and an “UST-9124 Intra Cavity transducer” (frequency range of 3.0 MHz.—7.5 MHz; Wiener Neudorf, Austria) and the FNPO was defined as described previously [4, 23], namely as all follicles with a diameter < 10 mm, counted by a slow scan across the ovary. The FNPO threshold for defining follicular excess was 12 follicles per ovary. It was possible to perform a transvaginal ultrasound in all women.

### Pulsatile GnRH treatment

**Fig. 1:**
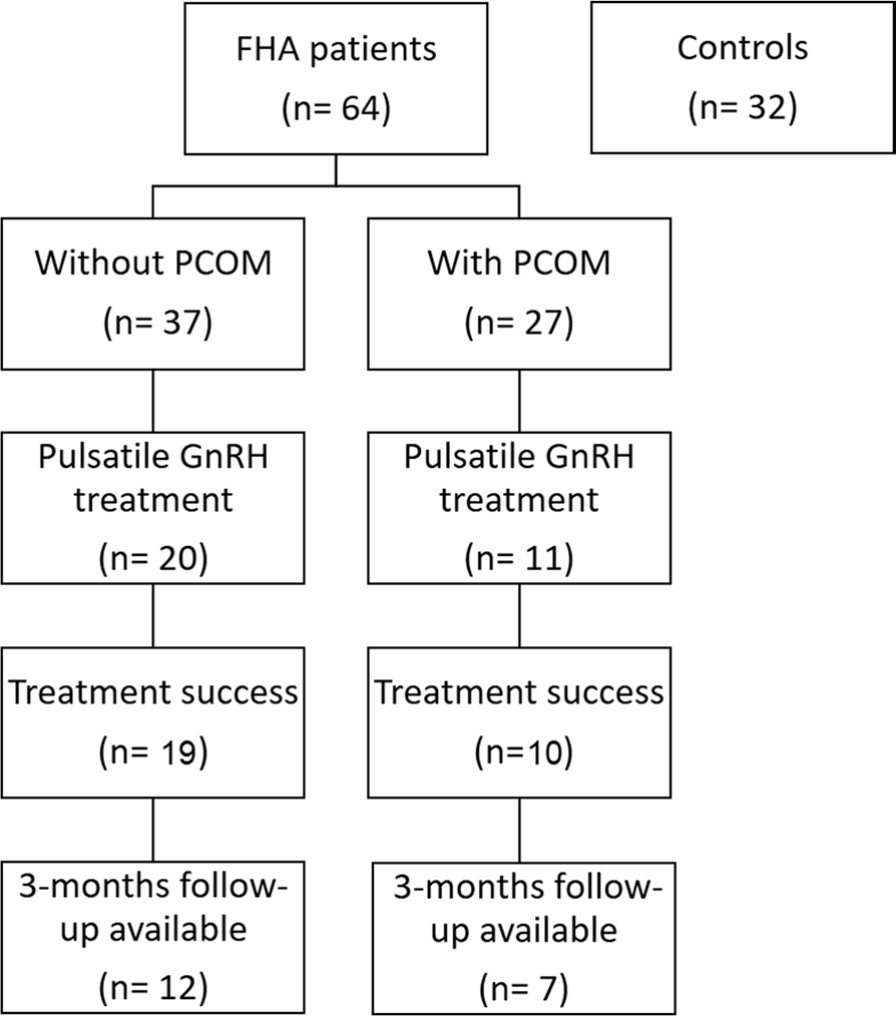
Study flow chart

From October 2019 on, subcutaneous pulsatile GnRH treatment was offered as first line treatment to the 31 FHA women seeking a pregnancy. Although it has never been recommended by any international guidelines to prefer pulsatile GnRH treatment over low dose FSH stimulation as a first line treatment, this regimen has been proposed by a recent review [24]. As demonstrated in Fig. 1 (study flow chart), serological follow-up parameters on cycle days 2–5 after the third menstruation were available in 19 (61%) after exclusion of one woman who did not tolerate the treatment (3.2%), one non-responder (3.2%) and ten women who achieved a pregnancy within the first three months of treatment (10/29, 34.5%).

Subcutaneous pulsatile GnRH therapy was given using a starting dose of 15 µg GnRH (LutreLef ®, Ferring Arzneimittel Gesellschaft m.b.H., Vienna, Austria) per pulse administered every 90 min subcutaneously via the LutrePulse system (LutreLef ®, Ferring Arzneimittel Gesellschaft m.b.H., Vienna, Austria) as described previously [25]. Women were instructed by a physician how to use the LutrePulse ® manager and pod systems. They had to change the pod every 72 h by themselves. All women underwent regular transvaginal ultrasound examinations to prove follicle maturation and ovulation.In addition, ovulations were documented by progesterone levels > 3 ng/mL on cycle days 19–23 [26].

### Statistical analysis

Due to the non-Gaussian distribution, continuous variables are presented as medians with interquartile ranges (IQR). The nonparametric Mann–Whitney u test was used to compare independent continuous variables, the Wilcoxon signed rank test was used to compare dependent continuous variables, and the chi square or Fisher’s exact tests were used to compare categorical variables between two groups. Between three groups, the Kruskal–Wallis test was used to compare independent continuous variables. Paired comparisons between 3 groups were performed by non-parametric analysis of variance (ANOVA) after rank transformation using the methodology suggested by Conover and Iman [27]. Univariate correlations between some variables were sought by Spearman’s non-parametric test. The IBM Statistical Package for Social Science software (SPSS 25.0) was used for all statistical tests. *P*-values < 0.05 were considered significant.

## Results

Causes for FHA, basic patient characteristics and results of hormonal testing in the FHA and the control groups are presented in Table 1. None of the FHA patients had ever been pregnant. Lower levels of FSH, LH, prolactin, and estradiol were found in women with FHA (*p* < 0.05).

**Table 1:** Basic patient characteristics and results of hormonal testing in women with FHA and healthy control women

	FHA (*n* = 64)	Controls (*n* = 32)	p
Underweight < 18.5 kg/m^2 a,b^	12 (18.8%)	-	-
weight loss > 10kg^a,b^	16 (25.0%)	-	-
Eating disorder^a,b^	19 (29.7%)	-	-
High level of exercise^a,b^	26 (40.6%)	-	-
History of emotionally stressful events^a,b^	15 (23.4%)	-	-
Age (years)^c^	26 (21;29)	26 (23;28)	0.551
BMI (kg/m^2^)^c^	20.0 (18.6;22.4)	21.5 (19.6;23.6)	0.064
TSH (IU/mL)^c^	1.67 (1.15;2.16)	1.41 (1.05;1.93)	0.186
FSH (mIU/mL)^c^	4.5 (3.2;6.2)	5.6 (4.4;6.7)	0.026
LH (mIU/mL)^c^	2.6 (1.3;5.3)	4.7 (3.6;6.7)	< 0.001
Prolactin (ng/mL)^c^	8.3 (6.6;12.5)	12.2 (9.4;14.0)	0.003
Oestradiol (pg/mL)^c^	24 (14;37)	59 (47;68)	< 0.001
Testosterone (pg/mL)^c^	0.22 (0.13;0.30)	0.24 (0.17;0.31)	0.479
Androstenedione (ng/mL)^c^	1.45 (0.98;2.37)	1.58 (1.19;2.04)	0.825
DHEAS (µg/mL)^c^	2.16 (1.53;3.06)	2.16 (1.80;2.74)	0.963
SHBG (nmol/L)^c^	73.2 (55.1;104.0)	81.2 (61.1;104.5)	0.397
AMH (ng/mL)^c^	2.57 (1.57;6.18)	3.08 (2.24;4.07)	0.960

Variables are expressed as ^a^numbers (frequencies) or.^c^median (IQR)

Multiple nominations are possible for ^b^FHA causes

In the FHA group, 27 women (42.2%) revealed PCOM on ultrasound, whereas 37 (57.8%) did not. Significant differences between the three groups (controls, FHA without PCOM and FHA with PCOM), were found for LH, prolactin, estradiol, and AMH levels (details are provided in Table 2).

**Table 2:** Basic patient characteristics and results of hormonal testing in FHA women with and without PCOM

	FHA without PCOM (*n* = 37)	FHA with PCOM (*n* = 27)	Controls (*n* = 32)	p
Age (years)	26 (21;30)	26 (21;28)	26 (23;28)	0.731
BMI (kg/m^2^)	20.0 (18.5;21.6)	20.4 (18.6;23.2)	21.5 (19.6;23.6)	0.131
TSH (IU/mL)	1.78 (1.17;2.04)	1.49 (1.08;2.36)	1.41 (1.05;1.93)	0.400
FSH (mIU/mL)	4.0 (2.6;6.1)	4.6 (3.5;6.5)	5.7 (4.4;7.6)	0.034
LH (mIU/mL)	2.6 (1.2;4.9)^b^	2.6 (1.3;6.3)^c^	4.7 (3.4;7.0)^b c^	0.001
Prolactin (ng/mL)	7.9 (6.5;12.2)^b^	9.3 (6.6;13.1)	11.8 (9.0;15.4)^b^	0.013
Estradiol (pg/mL)	23 (16;35)^b^	30 (11;45)^c^	71 (50;97)^b c^	< 0.001
Testosterone (pg/mL)	0.22 (0.13;0.30)	0.22 (0.15;0.30)	0.25 (0.17;0.31)	0.695
Androstenedione (ng/mL)	1.29 (0.93;2.10)	1.65 (1.09;2.62)	1.54 (1.18;2.04)	0.269
DHEAS (µg/mL)	2.01 (1.47;3.25	2.33 (1.56;2.84)	2.18 (1.76;2.74)	0.926
SHBG (nmol/L)	79.4 (60.3;120.9)	68.3 (37.7;99.8)	81.2 (61.;104.5)	0.144
AMH (ng/mL)	2.03 (1.40;2.50)^a b^	6.38 (4.34;10.10)^a c^	3.08 (2.24;4.10)^b c^	< 0.001

Variables are expressed as median (IQR); non-parametric Anovas with post hoc Bonferonni correction were perfomed

^a^significantly different between FHA without PCOM and FHA with PCOM

^b^significantly different between FHA without PCOM and controls

^c^significantly different between FHA with PCOM and controls

**Fig. 2:**
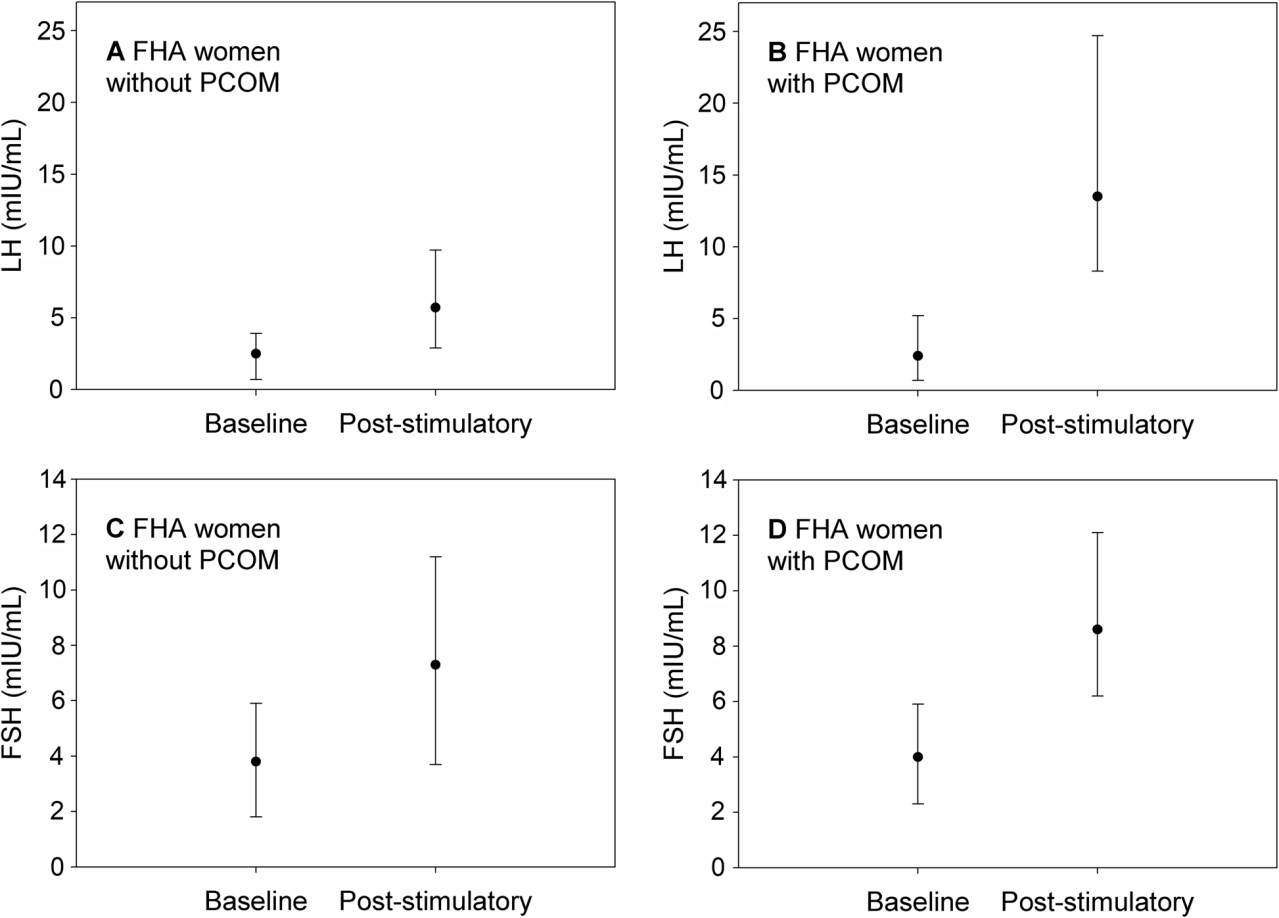
Dynamics in LH (**A**, **B**) and FSH (**C**, **D**) in FHA women with (**B**, **D**) and without (**A**, **C**) PCOM

Results of the GnRH stimulation tests were only available in the FHA group and are depicted in Fig. 2. Twenty minutes after stimulation, both LH and FSH increased significantly in FHA women without and with PCOM (*p* < 0.001). However, the median LH increase to 240.0% (IQR 186.4–370.0) in women without PCOM was significantly lower compared to the increase to 604.9% (IQR 360.0–1122.0) when PCOM was present (*p* < 0.001). In contrast, there was no difference between these two groups concerning the increases in FSH (194.2%, IQR 160.9–228.3, versus 209.4%, IQR 177.1–258.0; *p* = 0.345). An additional analysis revealed that basal AMH level was positively correlated with LH increase during the stimulation test (*r* = 0.455, *p* < 0.001; Supplementary Table).

FHA women without PCOM revealed significant positive correlations between basal FSH and AMH as well as between basal LH and AMH, whereas a significant positive correlation between basal LH and BMI was found in women with PCOM exclusively (*p* < 0.05). No correlation between age and serum AMH was seen in any of the two groups, but this was the case in controls (*r* = -0.254; *p* = 0.046). Moreover, in controls, basal serum LH was positively correlated with serum AMH (r-0.362, *p* = 0.042; Table 3).

**Table 3:** Correlation analyses in women with FHA and controls

	Patients without PCOM (*n* = 37)		Patients with PCOM (*n* = 27)		Controls (*n* = 32)	
	Correlation coefficient	p	Correlation coefficient	p	Correlation coefficient	p
Age:basal AMH	-0.139	0.411	-0.067	0.739	-0.254	0.046
Basal FSH:AMH	0.416	0.011	0.104	0.605	0.069	0.708
Basal LH:AMH	0.437	0.007	-0.010	0.959	0.362	0.042
Basal LH:BMI	-0.011	0.952	0.435	0.023	-0.011	0.952

Finally, 20 FHA women without PCOM received pulsatile GnRH treatment that was successful in 19 (95.0%) by means of regular ovulations and menstrual bleedings. Three-month follow up concerning hormonal levels was available for 12 patients (Table 4). In the group of FHA patients with PCOM, 11 women received pulsatile GnRH treatment and 10 (90.9%) responded successfully to it by achieving an ovulation. Three-months follow-up data were available in 7 of these women (Table 4). Only in FHA women without PCOM, AMH had increased significantly (baseline: median 1.69 ng/mL, IQR 1.58–2.20; after three months of treatment: 2.02 ng/mL, IQR 1.81–2.49; *p* = 0.002).

**Table 4:** Hormonal dynamics in FHA women with and without PCOM who responded to three months of pulsatile GnRH treatment

	FHA patients without PCOM (*n* = 12)			FHA patients with PCOM (*n* = 7)		
	Baseline	3-months follow-up	p	Baseline	3-months follow-up	p
FSH (mIU/mL)	5.1 (1.6;7.5)	6.9 (3.7;8.4)	0.002	4.8 (4.0;6.5)	5.0 (4.2;5.4)	0.933
LH (mIU/mL)	1.5 (0.3;4.9)	4.6 (3.8;6.3)	0.016	3.0 (0.6;6.9)	7.3 (6.6;9.1)	0.018
Estradiol (pg/mL)	21 (9;36)	46 (38;55)	0.005	24 (11;41)	56 (49;66)	0.028
AMH (ng/mL)	1.69 (1.58;2.20)	2.02 (1.81;2.49)	0.002	4.9 (3.6;7.4)	5.2 (3.6;6.3)	0.237

Additional correlation sub-analyses were performed, which can be seen in the Supplementary Table. In women with a three-months follow-up, basal AMH levels were negatively correlated with the FSH increase after pulsatile GnRH treatment (*r* = _0.731; *p* < 0.001) and positively correlated with the LH increase after pulsatile GnRH treatment (*r* = 0.520; *p* = 0.027). Moreover, the pre- to post-treatment dynamics in AMH were positively correlated with the dynamics in FSH (correlation coefficient 0.540; *p* = 0.017). A trend for this result was also seen in the sub-analysis of FHA patients without PCOM (*r* = 0.565; *p* = 0.055). In FHA women with PCOM, there was a positive correlation between basal AMH levels and the LH increase after three months of pulsatile GnRH treatment (*r* = 0.857; *p* = 0.014).

## Discussion

Our results confirm the recent findings of Makollé et al. [4], namely that serum AMH levels are low in women with FHA once patients with incidental PCOM are excluded. As hypothesized by Makollé et al. [4], and according to the two-triangle hypothesis [28], the reason for low AMH levels in FHA is the relative FSH deficiency that leads to a decrease in the pool of growing follicles and therefore to a decrease in ovarian AMH production. Here, we afford supplementary evidence to this hypothesis by showing a positive significant relationship between serum AMH and basal FSH levels. Even more convincing is our observation that in FHA women without PCOM, serum AMH increased significantly after 3 months of pulsatile GnRH treatment, along with basal serum FSH rise, close to significance (*p* = 0.055; Supplementary Table).

Our data about the relationship between serum AMH and LH levels differs however from those of Makollé et al. in whom the correlation between serum AMH and basal LH levels was not significant in FHA women without PCOM, contrary to controls [4]. To explain this finding, the authors hypothesized that the defects in neural systems involved in the pathophysiology of FHA, such as KISS/KISS1R or neurokinin [29], likely occur upstream of the stimulating effect of AMH on GnRH neurons [30]. Here we report a positive significant correlation in FHA women without PCOM as well as in controls. To reconcile this disagreement, it must be stressed that Makollé et al. included only patients with basal LH levels < 2 IU/L, i.e., with presumably more severe defects in neural systems than in our patients [4]. However, our patients had truly a LH deficiency, as indicated by their basal LH levels frankly lower than in controls and by their relatively modest increase during the GnRH stimulation test. Furthermore, in this sub-group, the AMH increase under pulsatile GnRH treatment tended to be positively associated to the LH increase. Altogether, these data are in agreement with the hypothesis of a stimulating effect of AMH on GnRH neurons and pituitary responsiveness to GnRH.

As in previously reported series [4, 8, 31–34], we found a high prevalence of FHA patients with PCOM at sonography (42%) that is higher than in the general population of similarly aged women. This observation remains unexplained so far. As recently reported for the first time by Makollé et al. [4], we found a positive correlation between serum AMH levels and BMI in this subgroup of patients exclusively. Makollé et al. speculated from this finding that women with FHA and PCOM had initially the PCOS-typical metabolic burden before losing weight, an assumption that was supported by the finding of lower SHBG levels in this group of patients [4]. This, however, does not directly explain the high rate of PCOM compared to a general female population of similar age. Thus, as already mentioned [4], one could hypothesize that women with PCOS or PCOM could be more prone to an inhibition of the GnRH neurons due to weight loss and other causes for FHA than women without PCOM. This would at least explain the over-representation of PCOM in women with FHA.

In addition to these considerations, the fact that basal LH and FSH levels were similar between FHA patients with or without PCOM (Table 2) together with the association between FHA and PCOM raises the issue of intra-ovarian dysregulation in PCOM that persists despite low gonadotropin levels. Epigenetic reprogramming in utero has been discussed as a plausible hypothesis recently. In detail, these modulations could affect granulosa cells by means of their richness and/or affinity of FSH and/or androgen receptors [35, 36]. In addition, such intra-ovarian factors could also explain why a decline in gonadotropins, which is typical for FHA (Table 1), would not interrupt the AMH excess in women with PCOS or PCOM, a hypothesis that has already been mentioned previously [4]. Otherwise, the discrepancy to the popular “two-triangle” [23] theory could not be explained.

Such an explanation at the intra-ovarian level could also be extrapolated to the pituitary, given the strong response of LH to a GnRH bolus that we observed in FHA patients with PCOM exclusively. This could be seen also as a sequelae of epigenetic reprogramming. Such pattern of LH response during the GnRH test (*i.e.*, strong elevation despite low basal level) has been known for long time in FHA but, to our knowledge, it is the first time that it is shown to be associated to PCOM. This data is in agreement with the observation by others that a similar pattern is observed in normal women with PCOM [11]. Interestingly, the LH response during the GnRH test was correlated to the basal AMH level in the whole group of patients with FHA, with a trend close to significance in those with PCOM, contrary to patients without PCOM. Furthermore, the basal LH increase after three months of pulsatile GnRH treatment was significantly correlated to pre-treatment basal AMH in patients with PCOM exclusively. These findings about the particular relationship between serum AMH and LH in women with FHA and PCOM are in reminiscence to those observed in women with genuine PCOS.

This raises the issue as to whether PCOM in FHA patients is merely a normal variant, as in the general population, or is part of a “hidden” PCOS in whom hyperandrogenism would have been abrogated by chronically low LH levels. The fact that FHA patients behave similarly under pulsatile GnRH treatment whether they have PCOM or not would favor the first hypothesis, as previously reported [9]. However, some authors reported that PCOS was unmasked by pulsatile GnRH therapy in a subgroup of women with FHA [37]. The two hypotheses are not mutually exclusive, as suggested by cluster analysis in patients with FHA, showing the following three clusters: one including patients without PCOM, one with mild features of PCOM, similar to controls with PCOM, and the last (the smallest) with more marked abnormalities as found in PCOS [7]. It might be the latest that correspond to hidden PCOS that could be unravelled by pulsatile GnRH therapy. We did not observe such an evolution in any of our patients but our series is too small to draw final conclusions on this issue.

The major strength of our study is the availability of data about the GnRH stimulation test and about hormonal follow-up data after pulsatile GnRH treatment study whose dependence to the presence or absence of PCOM have never been reported previously, to our knowledge. This allowed more complete insights about the influence of PCOM into the biological pattern of FHA. However, several limitations also have to be addressed. First, the retrospective design of our study; second, the lack of the precise follicle number per ovary that would have been an interesting parameter to report (it was recorded as a binary variable, i.e., either < or ≥ 12 follicles/ovary to define PCOM) and third, the lack of a sample-size calculation together with the comparably small sample size, especially in the subgroup analyses.

## Conclusions

In women with FHA without incidental PCOM, serum AMH levels are lower than in controls. In these FHA patients, AMH tends to rise after three months of pulsatile GnRH treatment and this seems to be associated with a rise in FSH, which underlines that the phenomenon of low AMH levels is based on relative gonadotropin deficiency rather than diminished ovarian reserve. Such findings have been reported in women with congenital hypogonadotropic hypogonadism under recombinant FSH [38]. Moreover, the finding of PCOM in women with FHA is rather frequent. In this subgroup of patients, there was a marked increase in LH levels after a GnRH stimulation test. This suggests differences in systemic and/or intra-ovarian regulation of ovarian function associated to the presence of PCOM.

## Supplementary Information

**Additional file 1:** **Supplementary Table.** Additional correlation analyses in women with FHA.

## Data Availability

The datasets used and/or analysed during the current study are available from the corresponding author on reasonable request.

## Abbreviations

ANOVA: Analysis of variance
AMH: Anti-Müllerian hormone
BMI: Body mass index
DHEA-S: Dehydroepiandrosterone-sulphate
ECLIA: Electrochemiluminescence immunoassays
FHA: Functional hypothalamic amenorrhea
FNPO: Follicle number per ovary
FSH: Follicle stimulating hormone
IQR: Interquartile range
LH: Luteinizing hormone
GnRH: Gonadotropin releasing hormone
PCOM: Polycystic ovarian morphology
PCOS: Polycystic ovary syndrome
SHBG: Sexual hormone binding globulin
TSH: Thyroid-stimulating hormone
