# Supplementary material for: Basal and dynamic relationships between serum anti-Müllerian hormone and gonadotropins in patients with functional hypothalamic amenorrhea, with or without polycystic ovarian morphology
Source: Reprod Biol Endocrinol. 2022 Jul 4;20:98. doi: 10.1186/s12958-022-00961-y (PMC9251918; doi:10.1186/s12958-022-00961-y)
Supplement: Supplementary file 1 — Additional file 1: Supplementary Table. Additional correlation analyses in women with FHA. [file 12958_2022_961_MOESM1_ESM.docx]

**Supplementary Table**. *Additional correlation analyses in women with FHA*.

|  | Correlation coefficient | p | Correlation coefficient | p | Correlation coefficient | p |
| --- | --- | --- | --- | --- | --- | --- |
|  | *All patients (n= 64)* | | *Patients without PCOM (n= 37)* | | *Patients with PCOM (n= 27)* | |
| Basal AMH:FSH increase after GnRH-stimulation | 0.212 | 0.092 | 0.176 | 0.298 | 0.164 | 0.415 |
| Basal AMH:LH increase after GnRH-stimulation | 0.455 | *<0.001* | 0.033 | 0.846 | 0.318 | 0.106 |
|  | *All patients (n= 19)* | | *Patients without PCOM (n= 12)* | | *Patients with PCOM (n= 7)* | |
| Basal AMH:FSH increase after three months of pulsatile GnRH treatment | -0.731 | *<0.001* | -0.446 | 0.146 | 0.429 | 0.337 |
| Basal AMH:LH increase after three months of pulsatile GnRH treatment | 0.520 | *0.027* | -0.183 | 0.591 | 0.857 | *0.014* |
| AMH increase:FSH increase after three months of pulsatile GnRH treatment | 0.540 | *0.017* | 0.565 | 0.055 | -0.107 | 0.819 |
| AMH increase:LH increase after three months of pulsatile GnRH treatment | 0.127 | 0.615 | 0.584 | 0.059 | 0.000 | 1.000 |
